# Human Milk Lipidomics: Current Techniques and Methodologies

**DOI:** 10.3390/nu10091169

**Published:** 2018-08-26

**Authors:** Alexandra D. George, Melvin C. L. Gay, Robert D. Trengove, Donna T. Geddes

**Affiliations:** 1 School of Molecular Sciences, The University of Western Australia, Crawley, Perth, WA 6009, Australia; alexandra.george@research.uwa.edu.au (A.D.G.); melvin.gay@uwa.edu.au (M.C.L.G.); 2 Separation Science and Metabolomics Laboratory, Murdoch University, Murdoch, Perth, WA 6150, Australia; R.Trengove@murdoch.edu.au

**Keywords:** human milk, breastfeeding, lactation, lipids, lipidomics, mass spectrometry, chromatography, NMR spectroscopy

## Abstract

Human milk contains a complex combination of lipids, proteins, carbohydrates, and minerals, which are essential for infant growth and development. While the lipid portion constitutes only 5% of the total human milk composition, it accounts for over 50% of the infant’s daily energy intake. Human milk lipids vary throughout a feed, day, and through different stages of lactation, resulting in difficulties in sampling standardization and, like blood, human milk is bioactive containing endogenous lipases, therefore appropriate storage is critical in order to prevent lipolysis. Suitable sample preparation, often not described in studies, must also be chosen to achieve the aims of the study. Gas chromatography methods have classically been carried out to investigate the fatty acid composition of human milk lipids, but with the advancement of other chromatographic techniques, such as liquid and supercritical fluid chromatography, as well as mass spectrometry, intact lipids can also be characterized. Despite the known importance, concise and comprehensive analysis of the human milk lipidome is limited, with gaps existing in all areas of human milk lipidomics, discussed in this review. With appropriate methodology and instrumentation, further understanding of the human milk lipidome and the influence it has on infant outcomes can be achieved.

## 1. Introduction

Human milk (HM) is vital to the infant, providing both immune protection and energy required for optimal infant growth. Breastfeeding is associated with multiple benefits for both the infant and the mother, such as decreased risk of asthma, pneumonia, type 1 diabetes, and obesity and decreased incidence of breast and ovarian cancer, respectively [1,2,3]. Further, these breastfeeding benefits increase with the duration of breastfeeding [1,4].

The macronutrient composition of HM consists of approximately 7% carbohydrates, 5% lipids, 0.9% protein, and 0.2% minerals emulsified in an aqueous milk matrix [5]. While the lipid portion of HM makes up only 5% of mature milk, it contributes to over 50% of the infant’s daily energy requirement [6]. These lipids are known to be involved in both neural and retinal tissue development as well as immune system development and defense in the infant [7,8,9]. Furthermore, the HM lipid profile impacts early growth in preterm infants [10].

Despite the importance of these lipids, the total lipid content in HM is highly variable, with large changes occurring throughout the day, between breasts, between women, and throughout the whole lactation period [11]. Interestingly, the total HM lipid content is not believed to be changed by maternal diet; however, diet influences the specific fatty acid (FA) composition. One example of this is docosahexaenoic acid (DHA)-containing triacylglycerides (TAGs) which have been found to be in higher concentrations in HM of women with high seafood intake [12,13]. The concentrations of DHA, docosapentaenoic acid (DPA), and arachidonic acid (AA) are also observed to decrease over the lactation period and these are the three FAs implicated in infant neural and retinal development [14].

Along with the variability of HM lipids, the complexity of the milk matrix and lipid hydrophobicity adds to the difficulty of a comprehensive lipidomic analysis. Further, over 40,000 biological lipid structures have been identified in various biological matrices such as human blood and plant material, leaving the possibility for thousands of lipids to be identified and deconvoluted in HM [15].

A number of basic analytical techniques have been employed over the years to investigate the lipid composition of HM; however, with the recent advancement of analytical techniques such as chromatography coupled with mass spectrometry and nuclear magnetic resonance spectroscopy, current analysis promises to be more comprehensive. Lipidomics is the research field in which complex lipidome analyses are carried out to produce a comprehensive and quantitative description of the lipid species present in a given matrix. While lipidomics is expanding exponentially in biological research, it is only recently being applied to HM. Lipids can be defined as FAs and their derivatives, or by their solubility in organic solvents and insolubility in inorganic solvents. Fat-soluble vitamins such as vitamin D are often included within this definition of lipids, but for the purpose of this review only standard lipid classes such as FAs, glycerolipids, glycerophospholipids, sphingolipids, sterols, prenols, which have been identified in HM will be discussed [16,17,18,19,20].

Additionally, this review will investigate the current status of HM lipidomic analysis and the new emerging techniques, methods, and instruments being used. It will focus on analysis of HM lipidome composition, rather than simply total lipids, which has commonly been estimated using creamatocrit or gravimetric methods [21]. With the present-day state of ‘omics’ techniques, the ability to comprehensively and quantitatively analyse the HM lipidome will allow a greater understanding of HM lipids. However, in order to make significant advances in HM analysis, quality control and standardised sampling must be routinely employed. Lipidomics platforms hold great promise to further elucidate HM lipid composition and the role of lipids with respect to infant health and disease.

## 2. Sampling

HM lipid content and composition, as mentioned above, is highly variable and constantly changing to meet the demands of the infant. The total lipid content of HM varies widely between women, throughout a feed, a day, and lactation, with reported values ranging widely from 11.4 g/L to 61.8 g/L [11,22,23]. While Jensen suggests that maternal age may influence HM lipid content, this has not been validated [11]. Similarly, diet has previously been suggested to influence lipid content, yet no studies exist to confirm this. In contrast, the FA composition of the lipids is influenced by maternal diet, where areas of China with high fish intake have significantly higher HM DHA than other provinces with lower fish intake, and DHA supplementation of breastfeeding women in Australia also led to an increase in HM DHA content [24,25]. While different ethnicity is thought to be another contributor to lipid composition variability, this too is most probably related to maternal diet. Other maternal health conditions, such as infections or metabolic diseases, have also been noted to reduce the total lipids in HM [6]. An obvious limitation to sampling protocols is that these studies are dealing with human participants, a mother feeding her infant, therefore sampling protocols should not negatively impact or interrupt infant feeding and sleeping patterns. Sampling protocols are non-invasive, involving expression of milk from the nipple either manually or using a breast pump. Differences between sampling methods and timing of collection of the sample may also contribute to complexity and variations within these results, therefore strict collection protocols should be implemented in order to obtain representative samples for HM studies. Details of the methods used in HM lipidomics studies, as well as the other methodology and identified lipids of existing studies, are summarised in Table 1.

### 2.1. Sampling with Respect to the Feed

Fat content increases as the breast is drained of milk, during a feed, therefore sampling pre-feed HM will give lower total fat content than mid- or post-feed samples [26]. Studies often do not take this into account and do not specify when samples are taken, often accepting random samples from nonspecified time points. Some studies will sample at a single time point with no further details, prescribed time points or will attempt to investigate feeds more thoroughly by collecting pre-, mid- and/or post-feed samples [27,28,29,30,31]. One frequently used sampling method to interrogate the entire feed is to drain the whole breast using a breast pump and then sample from the pumped milk [20,32,33]. However, as infants rarely drain the whole breast [34,35], this method will remove more milk from the end of the feed which is higher in fat content leading to an overestimation of the infant consumption [36].

### 2.2. Sampling over 24 h

As fat content increases with removal of milk from the breast subsequently the HM lipid content varies over a 24-h period, increasing from the first to the last feed of the day, higher in the evening than in the morning [37]. By sampling and test-weighing the infant before and after each feed in a 24-h period, milk production can be measured in addition to the actual amount of milk lipid ingested by the infant [38].

### 2.3. Sampling through Stages of Lactation

In general, the total HM lipid content increases throughout lactation, with Mitoulas et al. showing that lipids decrease from the first to second month but increase up to month 9 of lactation [26]. However, the mean amount of fat delivered to the infant remains constant as maternal milk production and infant intake changes across the months [26]. In order to account for the fat variations at different lactation stages, prescribed time points for sampling within a study, such as sampling on certain days (e.g., day 1, 14, and 42 post-partum) or sampling over a period of lactation (e.g., first 22–25 days of lactation) should be chosen, depending on the research question [13,29]. However, many studies either collect at different stages of lactation and pool their samples (such as [39]), or fail to mention when the samples are collected which makes comparison with other studies and understanding the lipidome difficult.

### 2.4. Ideal Sampling Routine

Due to these variations of both the total lipid content and lipid composition, lipidomic analysis at any given time has the potential to be very different. It is important that the aforementioned factors are all taken into account when sampling HM and that the study is defined in order to control these influences. This is rarely the case in HM studies, clearly outlined by the missing data in Table 2. We suggest defining the research question and then determining the appropriate samples in order to define and standardize sampling to minimize variables and confounding factors. Given that we know about the lipid variations at any given time, it is important that studies use sampling with 24-h test-weighing of the infant during breastfeeding (and expression) to provide more accurate interpretation of infant intake to determine the influence of these lipids on infant development [38]. This technique is not yet widely used but would greatly improve interpretation of research studies. Taking into account the published sampling methods, these are likely to contribute greatly to the large variation in reported lipids values [40].

## 3. Storage

As with lipidomic analysis of all biological samples, care must be taken to minimise lipolysis and lipogenesis during storage due to enzymes, such as lipase (bile salt-stimulated lipase and lipoprotein lipase), which are present endogenously in HM [58,59]. While immediate analysis of the lipidome is ideal to minimize any compositional changes by lipase activity, in reality this is not practical, therefore correct storage and sample preservation is imperative. Poor consideration of adequate storage affects the reproducibility and interpretation of HM study results and, as shown in Table 1, is something rarely considered in HM lipidomics.

### 3.1. Freezing

Maintaining the integrity of a HM sample is carried out by freezing samples at temperatures such as −20 °C, −70 °C or −80 °C. If the sample is not frozen adequately, endogenous lipases have the opportunity to cause lipid hydrolysis resulting in inaccurate and misrepresentative HM lipid content for measurement. Studies have shown that while freezing HM at −20 °C for 3 months resulted in a significant loss of lipids (up to 20%), storage at −70 °C or −80 °C stops enzyme activity within the samples and HM lipid integrity is best preserved [60,61,62]. Although one study showed major lipid loss in HM samples stored at −80 °C, Fusch et al. reported that this is likely an effect of poor experimental controls [63,64]. The duration of storage is not routinely reported in published studies but is obviously another factor affecting results. Another key factor is the number of freeze-thaw cycles that the sample underwent prior to analysis. In a study by Bitman et al., up to 20% fat loss was observed when HM underwent two freeze-thaw cycles, due to the resulting increase in lipolytic activity in HM during each of these cycles [65]. Therefore, steps during sample handling should be carefully planned such that all samples undergo the same number of freeze-thaw cycles.

### 3.2. Preservatives

HM has inherent antioxidant capacity to reduce and prevent oxidative degradation [66]. This degradation most commonly occurs in unsaturated fats, where the double bonds undergo cleavage by free radicals. In addition to freezing HM samples, antioxidant preservation of HM samples has also been used to maintain sample integrity. Phenol derivatives such as butyrated hydroxytoluene (BHT) have been used in previous studies to prevent lipid peroxidation [13,67]. BHT works by preferentially reacting with any oxygen present so that there is no opportunity for the lipids to be oxidatively degraded. There are currently no HM studies examining BHT efficacy for lipid preservation; however, studies of other biological samples such as red blood cells have used BHT with success, resulting in increased red blood cell FA preservation from 4 weeks to at least 17 weeks [68].

## 4. Lipid Extraction

Following appropriate HM sampling and storage for lipid analysis, sample preparation is essential to ensure accuracy and reproducibility of the results. For lipidomics analysis, mass spectrometry techniques, which will be discussed in Section Section 7.2, are commonly used. Therefore, clean-up steps such as liquid–liquid extraction and/or solid-phase extraction are essential to remove interferences such as proteins and sugars, as well as concentrate the lipids of interest. Sample preparation methods used in HM lipidomics studies are described in Table 1. Prior to lipid extraction, the sample must be homogenised, to ensure a uniform distribution of milk fat globules throughout the sample.

### 4.1. Liquid-Liquid Extraction

**Figure 1:**
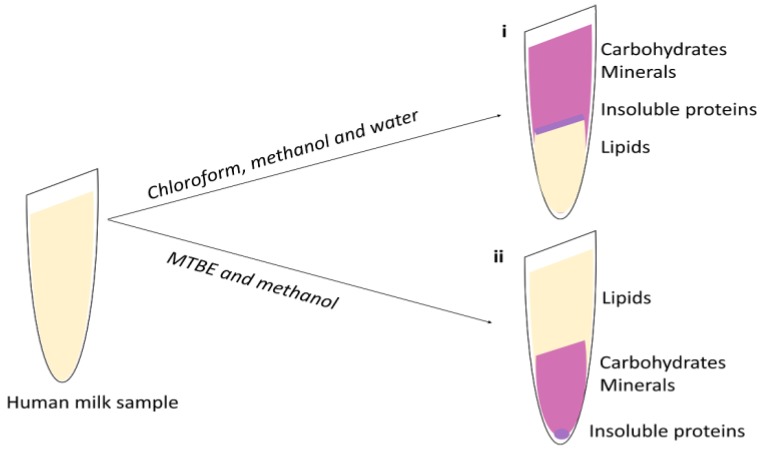
Liquid–liquid extraction of human milk lipids using (**i**) Folch extraction or (**ii**) Methyl-tert-butyl ether (MTBE) extraction.

Liquid-liquid extraction (LLE) techniques are used to separate analyses by their relative solubility in different immiscible liquids. LLE is the classical choice of lipid extraction method used in HM analysis, with variations of the 1950s methods such as Folch [14,69] and Bligh–Dyer [70], using chloroform, methanol, and water in ratios 8:4:3 and 1:2:0.8 respectively, being most commonly used. Other than the solvent ratio, the difference in these methods is that Bligh–Dyer uses smaller volumes of solvent and is a less time-consuming protocol [70]. While the Bligh–Dyer extraction was first developed on fish muscle, Folch extraction was developed on brain tissue, however both quoted as being easily adapted to other tissue types. When these solvents are added to HM, the lipids are dissolved into the organic phase (chloroform) and are separated from the aqueous phase (methanol and water, containing carbohydrates and salts) by a layer of cell debris and protein (Figure 1i).

While the use of these methods is well established, the drawbacks include the use of hazardous solvent, such as chloroform, and also the risk of contaminating or losing the lipid-containing lower phase when sampling through the aqueous phase or separating layers. These methods have been directly translated into HM studies or modified to either replace the use of hazardous solvent, such as chloroform with dichloromethane; or increase extraction efficiency with the introduction of centrifugation to enhance phase separation and the omission of water [27,29]. Recently a methyl-tert-butyl ether (MTBE) extraction method, initially developed for plasma lipid extraction, has been employed for HM lipid extraction for the analysis of both lipids and other HM metabolites [51]. This extraction, similar to the Folch and Bligh and Dyer method, separates lipids using phase separation. However, using the MTBE method, the organic phase containing lipids instead forms the upper layer, and the aqueous phase (containing the matrix pallet) forms the lower layer (Figure 1ii). This method has made extraction of lipids simpler and minimizes the potential of cross contamination.

### 4.2. Solid-Phase Extraction

**Figure 2:**
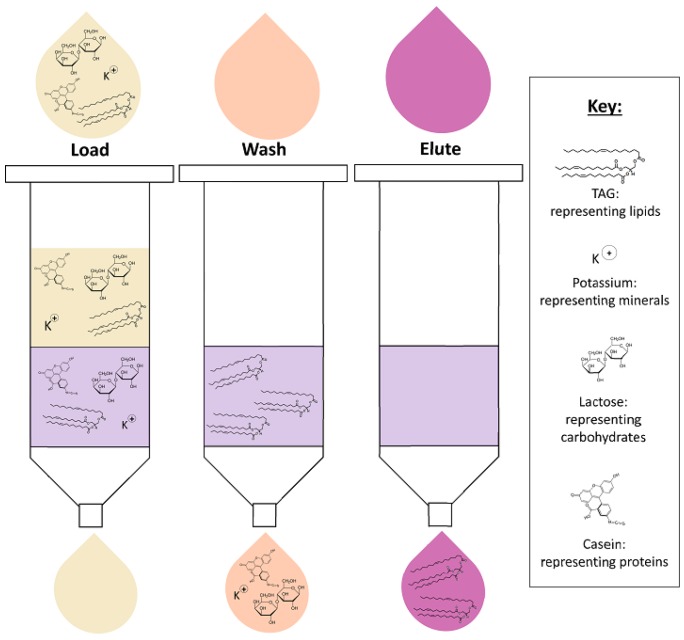
Solid-phase extraction of human milk lipids.

The use of solid-phase extraction (SPE), a type of column chromatography, is gaining popularity for its rapid and efficient lipid extraction from biological fluids. In this process, HM is loaded into the cartridge with lipid analyses retained on the solid-phase sorbent, such as C18, packed in a cartridge, meanwhile the interfering milk matrix components are washed out. Lipids can then be eluted from the bonded phase using organic solvents (Figure 2) [71]. Only two published milk lipidome studies have successfully used SPE for lipid extraction from HM, extracting fatty acyls, glycolipids, sphingolipids, prenol lipids and sterol lipids for analysis [46,55]. The first study by Dreiucker and Vetter uses a silver-ion SPE to extract FAs separating them by their degree of saturation and isomeric configuration [46]. The FAs were then eluted with acetone-based solvents, which then allowed better measurement of preseparated FA isomers by GC–MS than in standard LLE extraction. While this silver-ion SPE method is more quantitative, it has limitations with reproducibility and standardization to ensure complete lipid extraction. In another study, a solid-phase micro extraction (SPME) technique was used. This SPME involves the immersion of a solid-phase sorbent-coated fiber into HM and then use of organic solvent (such as isopropanol) to desorb the lipids [55]. This technique has poor reproducibility for the amount and type of lipids absorbed by the fiber, even when other parameters such as time and elution solvent are standardized, thus rendering this method suitable for qualitative analyses only. These factors limit the current use of SPE in HM lipidomics; however, further optimisation could offer the possibility of SPE automation in a plate format, which would make this technique ideal for routine, high-throughput extraction of HM for lipidomics.

## 5. Lipid Transesterification

**Figure 3:**
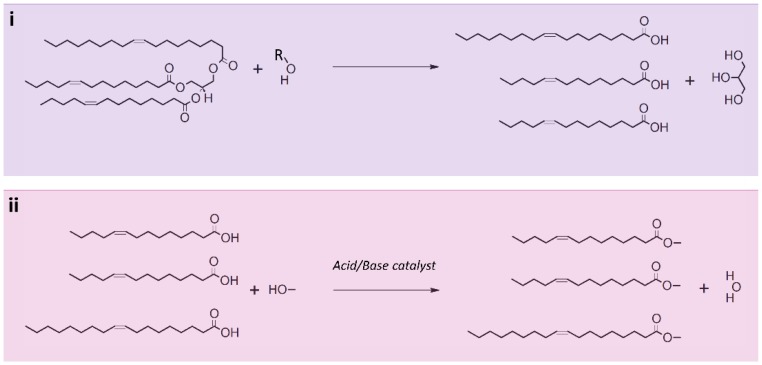
Transesterification reactions of triglyceride 14:1/14:1/18:1, one triglyceride commonly found in human milk. (**i**) Triglyceride hydrolysis, carried out with a base (such as KOH), resulting in glycerol and three free fatty acids; (**ii**) Resulting free fatty acid reaction with methanol and an acid/base catalyst producing three fatty acid methyl esters and water.

Following lipid extraction from HM samples, lipid transformation may be required for the analysis of non-volatile free or lipid-bound FAs. It is generally accepted that free FA in HM are artefacts of lipolysis, although only one study has investigated the FA from lipase hydrolysis of TAGs and other lipids (such as phospholipids and sphingolipids) [72]. This section will discuss only the analysis of FA that make up lipids, more specifically the FA composition of TAGs, which make up 98% of the lipids in HM, despite the methodology being poorly described (Table 1) [73]. Prior to the analysis of these FA, a two-part chemical transesterification is carried out, first hydrolysing the TAG, releasing three FA (Figure 3i), followed by derivatisation of the resulting FA to methyl esters (FAMEs) for GC analysis (Figure 3ii). This reaction can be either acid or base catalyzed. Derivatisation of FA is necessary for GC analysis as the high polarity of nonderivatised FA can result in hydrogen bond formation and therefore adsorption issues on a GC column, leading to band broadening and retention time shifting [74]. The resulting FAMEs have reduced polarity, able to be separated by a polar GC column.

The transesterification method is well-established and has been widely applied in FA analysis, where acidic transesterification using boron trifluoride (BF_3_) is most commonly used, as first described in 1964 [75]. The early HM FA transesterification methods frequently use this BF_3_ and methanol approach [10,12,13,43,48]. In other HM studies, transesterifications have used acid catalysis (methanolic-hydrogen chloride) or base catalysis (using methanolic-potassium hydroxide or sodium-methoxide) [19,32,45,72]. Although BF_3_ is a hazardous chemical and could also interact with BHT preservatives in a sample, it is still widely used in HM preparation [32,76]. The primary drawbacks of transesterification for FAME analysis are the laborious and time-consuming steps involved, supporting the movement towards methods not involving such preparations (such as liquid chromatography–mass spectrometry).

## 6. Quality Control

The use of quality control (QC) is essential to minimize influences, such as sample matrix effects and instrument variations that could cause issues with method accuracy and reproducibility. Despite the importance of QC in lipidomics, it is often overlooked in almost all, not just in HM, studies (as can be seen in Table 1). The QC measures are generally determined by several factors including the target lipid class of the study, the availability and cost of the standards and researcher preference. Several types of QC, which we have categorized as ‘in-sample’ and ‘out-of-sample’ QCs, should also be in place when lipidomic analyses are carried out and these are described below.

### 6.1. In Sample

This QC is added in known concentrations to HM during sample preparation and is also referred to as the internal standard (IS). For optimal lipidomics, more than one compound should be used as an IS. If these IS are added to HM prior to extraction, they can be used to assess variability that may occur in sample storage and extraction recovery. If the IS is added after sample extraction, it is used to monitor instrument performance and variability. The compound selected as an IS should be a labelled compound that is, or behaves as, the compound/s of interest. Due to limited availability of expensive commercial labelled lipid standards, to date no HM lipidomics studies have used labelled lipid standards. HM studies have, however, used a variety of unlabelled commercial lipids which are presumed not to be present in HM as an IS, for example heptadecanoic acid (C17:0) [28].

### 6.2. Out of Sample

QC samples should also be analyzed periodically within an experiment to monitor for any instrument abnormalities, such as sample degradation or loss of response. QCs are typically a pooled QC or commercial QC. A pooled QC is prepared by pooling aliquots of HM samples from the laboratory and analyzing these alongside a batch of samples. These QCs need to be rigorously prepared and stored in order to achieve reproducibility and for accurate monitoring of intra- and inter-batch variations. The pooled QC is the simplest and cheapest to prepare. Commercial QCs are known lipid analytes purchased to be run within a batch, like other out-of-sample QCs, confirming and identifying the retention time, *m*/*z* values and identity of these analyses. Additionally, these are often used to test an instrument for suitability. Out-of-sample QC should always be matrix matched to account for biological matrix effects, a condition that no HM lipidomic studies have yet met [77].

While there is currently no general consensus on the type of QC that should be used and the limits of variability within a lipidomics experiment, many studies will predefine the limits based on experience and the instruments used. Because of the vast number of lipids, untargeted HM lipidomics can only ever be semiquantitative [77].

## 7. Analytical Instrumentation for Lipidomic Analysis

Due to the complexity of lipids, complete lipidomic analysis requires more than one instrument platform. The choice of instrumentation for HM lipidomics therefore depends upon the study aims and the lipids of interest. Simple separation techniques have previously been used for qualitative analysis of lipids, such as thin-layer chromatography and gas chromatography (GC). Although GC is thought of as the gold standard for HM FA lipidomics, the availability and increasing prevalence of other separation techniques such as liquid chromatography (LC) and high-resolution mass analyzers, such as time-of-flight and Fourier Transform, means that the HM lipidome can be more comprehensively characterized [78]. The advantages and disadvantages of the instrumentation used in HM lipidomic analysis are summarized in Table 3. Consistent GC use in HM lipidomics can be seen in Table 1, with the slow emergence of mass spectrometry in recent years.

### 7.1. Separation Methods

#### 7.1.1. Gas Chromatography

GC coupled with a flame ionization detector (GC–FID) is the most routinely used separation method for FA analysis since the 1950s and is widely accepted for quantification of FA in many sample types, including HM [19]. Cyanopropyl-based columns ranging from 30 to 60 m in length are typically employed for FAME analysis. However, longer columns (up to 100 m) are used if separation of dietary FAME isomers such as *cis* C18:1 and *trans* C18:1 is desired. Therefore, the requirement for a longer GC column can extend both the method preparation and run time. The FID is generally used in FAME analysis as it is considerably cheaper to purchase and maintain compared to mass spectrometry (MS) detectors. Furthermore, the robustness of the FID allows the analysis of large numbers of samples before the need for any maintenance and does not have the same requirements and issues as MS (such as ionization source cleaning and ionization issues, as seen in mass spectrometry), discussed in Section 7.2. Mass Spectrometry [79]. Additionally, HM FAME analysis using GC is well characterized based on elution order and retention time, either requiring a limited number of standards or using Kovats retention index, as described in a HM study by Villasenor et al., for identification by comparing experimental and established retention indices [51]. Further, retention time locking can add to method reproducibility. However, GC–FID lacks mass selectivity, unlike MS, so it has been known to misidentify FAMEs in the presence of co-eluting compounds or contaminants that may be present in the sample, although this has not been investigated in HM studies [80,81].

#### 7.1.2. Liquid Chromatography

While GC is widely used for FA analysis, LC, with an evaporative light-scattering detector (ELSD), charged aerosol detector (CAD), electrochemical detector, or coupled to mass spectrometry (MS), has been used in the analysis of intact lipids, such as TAGs and phospholipids [79]. Currently only one study has used LC–ELSD in HM lipidomics, to quantify phospholipids, while other LC methodology is most commonly carried out using mass spectrometry [20,53]. Due to the wide variety of lipids in HM, various stationary phases and solvent combinations are employed depending on the type of lipids and separation required. Lipid separation in biofluids, including HM, is most often carried out using a C18 stationary phase column but other silica-based stationary phases, such as C8, have also been used in HM analysis for separation of all lipid classes and phospholipids, respectively [20,51]. Reversed-phase LC separates intact lipids and free FA based on their specific FA polarity, degree of saturation and chain length, while normal-phase LC will separate lipids, such as glycerophospholipids, by their class [82]. In LC analyses, the solvent and stationary phase must be compatible with the detection method, for example MS, where ratios of organic and inorganic solvents such as acetonitrile, alcohol, and water are most commonly used. When MS is the chosen detector ammonium salts (formate or acetate) and formic acid will be added (discussed in Section 7.2. Mass Spectrometry). The main advantage of LC over GC is that transformation is not required and intact lipids such as triglycerides can be analyzed [83].

#### 7.1.3. Supercritical Fluid Chromatography

Supercritical fluid chromatography (SFC) is another separation technique similar to LC, which, instead of using a liquid mobile phase, uses a supercritical fluid, such as carbon dioxide (CO_2_), as the mobile phase. Supercritical fluids are formed when dense compressed gas is subjected to a specific pressure and temperature. CO_2_ is the most commonly used supercritical solvent and its non-polar properties make it ideal for separating non-polar lipids like TAGs, shown by Laakso and Manninen in cow’s milk, to separate TAGs by their molecular size [84]. Although SFC has been widely used in dairy milk fat research and oil separation, its use in HM is limited to one study where SFC was coupled to mass spectrometry [57]. Advantages of SFC include no requirement for derivatisation, and the ability for SFC to be coupled with all detector types, such as FID or MS, as well as its low cost and waste output relative to LC, using less organic solvents than LC, and allowing faster separation and higher resolution than LC and GC in metabolomics analyses [85]. These features all make SFC well suited to the analysis of multiple lipid classes in one sample that have a range of polarities [79].

#### 7.1.4. Thin-Layer Chromatography

Like LC, thin-layer chromatography (TLC) may be qualitatively analytical but is more commonly used as a preparative step in human studies. HM studies often use TLC for separation of lipids into their individual classes, for example separation of short- and long-chain FAs prior to analysis [72]. This inexpensive technique is classically carried out using a silica plate and non-polar solvent for lipid class separation, and the classes can then be collected and analyzed using platforms such as GC or LC. As TLC does not have the separating resolution of GC or LC, its ability to perform identification is limited and thus may be the reason why TLC is not frequently used in HM lipidomics.

### 7.2. Mass Spectrometry

Mass spectrometry (MS) is the detection technique that identifies ionized compounds based on their mass-to-charge ratio (*m*/*z*). This is a destructive technique in which the sample is destroyed and cannot be used for future analysis. In HM lipidomics analysis, various types of mass analyzers, such as quadrupole, triple-quadrupole and time-of-flight, have been employed to identify and quantitate different lipids [79]. Given the increased sensitivity and specificity of MS in contrast to other detector types, such as FID and ELSD, it is possible to confirm the identity of known lipids, identify unknown lipids and to elucidate structural information of lipids using MS.

In order for lipids to be detected by MS, the compound needs to be ionized first using one of a variety of ionization techniques such as EI (electron ionization), ESI (electrospray ionization), CI (chemical ionization) or MALDI (matrix assisted laser desorption/ionization), which have been extensively reviewed [78,82]. These ionization methods can be carried out in either positive (EI, CI, or ESI) or negative (CI or ESI) mode, producing cations or anions respectively. In HM lipidomics, EI and ESI methods are commonly used. The EI technique is commonly used in conjunction with GC separation for FA analysis, where lipids are bombarded with a high-energy electron beam causing them to be ionized and fragmented in characteristic patterns. This is a hard ionization technique and generally only the fragment ions are observed [82]. Three HM studies have employed GC–MS since 2011, identifying and quantifying a large number of FAs as derivatised FAMEs, with MS having the added advantage of identifying many glycerolipids, glycerophospholipids, sphingolipids, prenol lipids, and sterol lipids not previously identified using GC–FID [46,50,51]. In contrast to EI–MS, ESI–MS is widely used in LC for HM lipidomics analysis [10,29,51,55]. This soft ionization technique involves pushing samples through a capillary with a voltage applied to it, creating a fine aerosol where ions are formed by desolvation. As ESI is a soft ionization technique, it is able to provide information on both the molecular ion (intact lipid, such as a triglyceride) as well as additional structural information by fragmenting the molecular ion, such as the FA composition of a specific triglyceride [82].

Additionally, LC–MS often uses additives such as ammonium formate and formic acid in the mobile phase as modifiers to promote ammonium adduct formation, these adducts being more stable than hydrogen adducts and easier to fragment than metal ions, and prevent retention time shifting [83]. The use of both positive and negative ionization mode in ESI–MS covers even more lipids, for example, identifying FAs using negative mode and phospholipids using positive mode [10].

Shotgun MS, which involves introducing a sample directly into the ion source and carrying out both positive and negative ionization mode MS, is a common technique for untargeted identification and structural characterization of lipids having been recently used for HM [52]. While this method is fast, sensitive and only requires a small amount of sample to be injected, the lack of chromatographic separation and ion suppression makes interpretation difficult. Ion suppression is a common effect where the response of a species of interest is suppressed due to endogenous matrix species such as proteins, or exogenous species such as plasticizers from plastic tubes/tube caps, in the sample compete for ionization [77]. This can be minimized with efficient lipid extraction during sample preparation, resulting in a cleaner and purer lipid extract. As lipid mixtures are challenging to interpret, chromatographic preseparation (GC or LC) is usually employed to further assist in separating lipids/isomers, providing additional orthogonal data for easier identification and more accurate quantification compared to the shotgun approach [79]. Additionally, untargeted analysis results in a large number of compounds to interrogate and often requires very specialized and expensive software.

### 7.3. Nuclear Magnetic Resonance Spectroscopy

Since the introduction of nuclear magnetic resonance spectroscopy (NMR) to the world of metabolomics, it has been used frequently in analyses of various biofluids and tissues, including muscle tissue and milk (such as in cows and camels) [86]. NMR is widely used in HM metabolomics to measure sugars, amino acids, and nucleotides; however, only one lipid (sn-glycero-3-phosphocholine), 12 phospholipid classes and a small number of lipid derivatives have been identified in HM by NMR [86,87]. NMR uses atomic magnetic properties, detecting every hydrogen/carbon/phosphorus-containing molecule and has the ability to provide valuable structural information for the intact lipid, such as structural differences between intact phospholipids [62,79]. In contrast to MS, NMR is a non-destructive technique, samples can be re-analyzed with NMR or other techniques [88]. However the drawbacks of NMR include signal overlapping, which can make discrimination of resonances from complex samples difficult, as well as larger sample volume requirements. While the use of NMR may be limited by its lower sensitivity than MS, NMR is highly reproducible and simple for a trained user to run [56]. Sample preparation may involve lipid extraction, such as with Folch extraction method, or simply whole milk may be analyzed. While preparation is simple, it can be difficult to run large numbers of samples with the same high-throughput capability of MS methods, unless an autosampler is available. The detected analyses can then be quantitated using the direct relationship between intensity of resonance and concentration [89].

## 8. Limitations and Future Perspectives

In addition to lipids being the most variable portion of HM, lipidomic analyses are limited by the number of samples analyzed, limiting the conclusions and relationships that can be identified in studies. Further, HM lipidomics would greatly benefit from standardized workflows for sample collection and preparation, analytical methodology on a wide number of platforms, data acquisition and data processing. The future of HM lipidomics needs higher lipid coverage on multiple platforms, allowing development of a HM metabolome/lipidome database similar to that of the Human Metabolome Database [90].

## 9. Conclusions

HM lipids are an essential macronutrient for the growth, development, and health of the infant; therefore, HM lipidomics are essential to provide a deeper understanding of short- and long-term infant health. The recent advances in instrumentation and methods in lipidomics will result in more comprehensive HM lipidomic investigations. Chromatography, MS, and NMR methods also offer potential for further lipid identification, structural elucidation, and investigation in HM. To develop better knowledge of the lipid changes in HM throughout lactation, more rigorous studies need to be carried out, employing stringent sampling and storage routines and advanced methodology with strict quality control. Rigorous protocols in HM investigations will allow more accurate assessment and investigation of the HM lipidome and the impact these lipids have on the infant.

## Figures and Tables

**Table 1:** Summary of existing human milk (HM) lipidomics studies from 1959 to 2018, including HM sampling, storage, preparation, quality control (in- and out-of-sample) and instrumentation used (- indicates not reported).

Lipids Identified	Sampling	Storage	Sample Preparation	Quality Control	Instrumentation	Reference
Fatty acids ranging from 10:0 to 22:6, including some unknown at the time	6 hospital participants, mid-feed samples (for 24 hours, pooled); 5 participants at home, random samples	4 °C (prior to pooling); −15 °C	1 or 2 mL human milk ➔LLE 95% ethanol-ethyl ether ➔Hydrolysis 5% methanolic-KOH ➔Derivatisation 5% methanolic-HCl	In: - Out: -	GC–FID Reoplex 400/Apiezon M column (Carrier gas: nitrogen)	Insull et al. (1959) [19]
Fatty acids ranging from 12:0 to 22:6	15 participant random samples (pooled)	-	4 mL human milk ➔TLC pre-separation ➔LLE chloroform:methanol (9:1) ➔Derivatisation BF_3_	In: - Out: -	GC–FID, 50 m CP-Sil-88 column (Carrier gas: nitrogen)	Haug et al. (1983) [27]
Fatty acids ranging from 6:0 to 26:0	7 participants, sampled on day 20–22 (mid-feed)	On ice ≤2 hours; 20 °C	-	In: C17:0 Out: -	GC–FID	van Beusekom et al. (1993) [28]
Polyunsaturated fatty acids ranging from 18:2 to 22:6; total saturated FAs; total monounsaturated FAs	23 participants 7-day samples from a single feed at weeks 6, 16, 30 (each time-point pooled)	−20 °C prior to delivery to laboratory	- mL human milk ➔Extracted - ➔Derivatisation 1% methanolic-H_2_SO_4_	In: - Out: -	GC 50 m BPX-70 column	Makrides et al. (1995) [14]
Fatty acids ranging from 10:0 to 22:6 including *cis* and *trans* isomers and some unknown at the time	198 samples, 3–4 weeks, mid-feed for a day (pooled)	-	5 g human milk ➔LLE chloroform:methanol (2:1) ➔0.02% BHT preservative ➔Derivatisation methanolic-BF_3_	In: Triheptadecanoin (in extraction solvent) Out: -	GC–FID 100 m SP-2560 column (Carrier gas: hydrogen)	Chen et al. (1995) [41]
Fatty acids ranging from 10:0 to 22:6	Samples from 84 participants at day 3 and weeks 2, 4, and 6	−20 °C	2 g human milk ➔LLE chloroform:methanol (2:1) ➔Derivatisation methanolic-BF_3_	In: Triheptadecanoin (in extraction solvent) Out: -	GC–FID 100 m SP-2560 column (Carrier gas: hydrogen)	Chen et al. (1997) [12]
31 Triglycerides	Pre- and post-feed samples from 11 participants between days 1–3, days 7–10, days 25–60 (47 samples)	−80 °C	1.5 mL human milk ➔LLE dicholoromethane-methanol (2:1)	In: C33:0 (after extraction) Out: -	LC–LSD, 250 mm Spherisorb ODS-2 column (Solvents: acetonitrile, dichloromethane, acetone)	Pons et al. (2000) [42]
Fatty acids ranging from 14:0 to 22:6	34 participants, samples on days 1, 4, 7, 14, 21, 28, at any time of day	−20 °C	≤2 mL human milk ➔LLE chloroform:methanol (2:1) ➔BHT preservative ➔Derivatisation methanolic-BF3	In: - Out: -	GC	Scopesi et al. (2001) [43]
Fatty acids ranging from 14:0 to 22:6	18 participants, days 1, 2, 3, 4, 5, 6, 7, 14, 28 between 0800–1000	4–8 °C (for <4 hours), deep freeze, 1 freeze-thaw cycle	100 µL human milk ➔LLE chloroform:methanol ➔Derivatisation –	In: Pentadecanoic acid Out: -	GC–FID 40 m Cyanopropyl DB-23 column	Minda et al. (2004) [44]
1. Fatty acids ranging from 4:0 to 22:6 2. 18:1 *t* isomers	81 samples, from complete breast expression, between 0600 and 0800 in the first month	Room temperature (4 hours); Lipid layer frozen at −20 °C	2 g human milk lipid layer ➔LLE chloroform:methanol (2:1) ➔Derivatisation sodium methoxide	In: - Out: -	1. GC–FID 100 m CP-Sil-88 column 2. GC–MS DB225 MS column	Mosley et al. (2005) [45]
Groups of FAMES and approximately 36 × specific FAMEs	1 random sample	−20 °C	1 mg human milk fat ➔LLE cyclohexane/ethylacetate, ➔Hydrolysis methanolic-KOH ➔Derivatisation BF_3_ ➔SPE fractionation Ag^+^-SPE	In: 14:0 and 17:0 Out: -	GC–EI–MS, 60 m SP2331 cyanosiloxane column (Carrier gas: helium)	Dreiucker et al. (2011) [46]
DHA and AA and other fatty acids	52 participants	-	1 mL human milk ➔Hydrolysis methanolic-KOH ➔Derivatisation H_2_SO_4_ ➔LLE hexane	In: C19:0 Out: -	GC–FID 50 m fused-silica CPSIL88 column (Carrier gas: helium)	Kelishadi et al. (2012) [47]
Fatty acids from 12:0 to 18:2	101 participant random samples over 3 days	−80 °C	20 µL human milk fat ➔Transesterification methanolic-BF_3_	In: Tridecanoic acid (in extraction solvent) Out: -	GC 100 m HP88 column (Carrier gas: helium)	Akmar et al. (2013) [48]
Total saturated and unsaturated fatty acids, 18:2 *n*6, 18:3 *n*3, 20:4 *n*6, 22:6 *n*3	29 mid-feed samples (8–12 weeks post-partum) between 1200 and 1500	−80 °C	100 µL human milk ➔Hydrolysis methanolic-NaOCH3 ➔Derivatisation methanolic-BF_3_	In: - Out: -	GC–FID 40 m RTX-2330 (Carrier gas: helium)	Saphier et al. (2013) [49]
Free fatty acids between C10 and C24	23 term and 15 preterm participants/38 post-feed samples during days 0–7 day, 8–21, >21	Frozen	500 µL human milk ➔LLE chloroform methanol ➔transesterification methanolic-HCl	In: C17:0 Out: -	GC–MS 30 m Ultra Alloy-5 column (Carrier gas: helium)	Chuang et al. (2013) [50]
Fatty acids between 4:0 and 22:6	50 participants 4 weeks post-partum, provided one full breast expression	−80 °C	250 µL human milk ➔Transesterification methanolic-HCl	In: 11:0 FAME, 13:0 TAG Out: -	GC–FID 100m CP-Sil 88 column (Carrier gas: hydrogen)	Cruz-Hernandez et al. (2013) [32]
Phospholipid classes	50 participants, pre-, mid-, post-feed samples at 4 weeks	−80 °C	250 mg human milk ➔LLE chloroforom:methanol (2:1) ➔Filtration PTFE filter	In: Phosphatidylglyceol Out: -	NP HPLC (ELSD) 2 x 250 mm Nucleosil 50-5 columns (Solvents: acetonitrile/methanol) NMR	Giuffrida et al. (2013) [20]
Polar and lipidic metabolites Tentative 287 lipids (positive mode), 126 lipids (negative mode)	52 samples between days 1 and 76, pooled. 10 participant samples at week 1, 9 participant samples at week 4	−80 °C (long term) −20 °C (short term)	50 µL human milk ➔LLE MTBE ➔Transesterification methanolic-HCl, BSTFA	In: C18:0 after extraction Out: Pooled HM	GC–Q–MS 30 m 122-5332G DVB5-MS column (Carrier gas: helium) LC–QTOF–MS (ESI) 15 cm EC-C8 column (Solvents: methanol water)	Villasenor et al. (2014) [51]
1. Fatty acids between 10:0 and 20:4 2. Triglycerides between 32:0 and 54:5	2 samples 4 random weeks post-partum	-	200 µL human milk ➔LLE (1) chloroform:methanol (2:1) ➔Transesterification with acid ➔LLE (2) chloroform: methanol: isopropanol (1:2:4)	In: 17:1–17:1–17:1 TAG, 17:0–14:1 PE, 17:0–14:1 PS, 17:0–14:1 PI, 18:1;2/17:0 SM (after extraction, for MS/MS) Out: -	1. GC–FID 60 m TRFRAME column (Carrier gas: helium) 2. MS/MS Triple TOF (positive and negative mode)	Sokol et al. (2015) [52]
Over 40 triglycerides	15 between-feed samples over days 1–5, 6–15 and >16		150 uL human milk ➔dichloromethane:methanol (2:1) ➔BHT preservative	In: - Out: -	HPLC–APCI–MS 150 mm Kinetex C18 column (Solvents: acetonitrile/n-pentanol)	Ten-Domenech et al. (2015) [53]
Fatty acids ranging from 10:0 to 22:6	477 participants gave pre-feed samples on days 1, 14, 42 between 1000 and 1100	−20 °C; −80 °C	200 µL human milk ➔LLE chloroform: methanol (1:1) ➔BHT preservative ➔Hydrolysis methanolic-KOH ➔Derivatisation methanolic-BF_3_ ➔SPE Sep-pak silica column	In: - Out: -	GC–FID 60 m DB-23 Fused silica column (Carrier gas: nitrogen)	Jiang et al. (2016) [13]
8 long-chain polyunsaturated fatty acids	514 participants, between 0900 and 1100 for first 22–25 days	−80 °C	0.2 mL human milk fat ➔Transesterification methanolic-CH_3_COCl	In: C17:0 Daturic acid Out: -	GC–FID 100 mm SP2560 column (Carrier gas: nitrogen)	Liu et al. (2016) [54]
1. Identified putative DHA-TAGs 2. Verified 56 DHA-TAGs ranging from C_45_H_74_O_6_ to C_67_H_116_O_6_	1 sample	-	0.2 mL human milk ➔LLE chloroform:methanol (2:1)	In: - Out: -	1. LC–ESI–triple quadrupole MS 250 mm synergi polar RP column 2. LC–ESI–LTQ–ORBI MS 2x 150 mm Poroshell 120 EC-C18 (Solvents: acetonitrile/water)	Liu et al. (2016) [29]
Polyunsaturated fatty acids	225 participants, provided pre- and/or post-feed milk at their own discretion, at 2 months	4˚C (≤24 hours); −80˚C	200 uL human milk ➔Transesterification -	In: - Out: -	GC–FID	Rosenlund et al. (2016) [30]
Groups of fatty acids, Glycerophospholipids, Prenol lipids, Glycerolipids, Sphingolipids, Sterol lipids	1 participant provided samples, at 1 year	−80˚C	1 mL human milk ➔SPME C18, isopropanol elution	In: - Out: -	LC–ESI–QTOF–MS 50 mm SB-C18 column (Solvents: methanol, water, hexane, isopropanol)	Garwolinska et al. (2017) [55]
sn-glycero-3-phosphocholine (and other lipid derivatives)	37 mothers provided 15 (morning and evening) samples on days 9, 12, 24, 31, 60, 85, 86, 87	−20˚C (2-8 days); −80˚C	- mL human milk ➔LLE methanol:water	In: - Out: -	NMR	Wu et al. (2016) [56]
64 Triglycerides ranging from C_33_H_62_O_6_ to C_65_H_120_O_6_	27 participants provided a day 7 and day 42 sample	−20˚C	0.1 mL human milk ➔LLE hexane ➔Filtration 0.22 µm nylon filter	In: - Out: 4 commercial QC 18:2/18:2/18:2; 18:1/18:1/18:1; 16:0/16:0/16:0; 18:1/16:0/18:1 for calibration curves	SFC ESI–QTOF 100 mm BEH-2-Ethylpyridine column (Solvents: supercritical CO_2_, methanol, acetonitrile)	Tu et al. (2017) [57]
Fatty acids ranging from 8:0 to 20:3	26 participants, left and a right sample at the same time on 3 consecutive days	−20˚C (≤1 week); −80˚C	- mL human milk ➔LLE chloroform:methanol (2:1) ➔Transesterification methanolic-H_2_SO_4_	In: - Out: -	GC–FID 50 mm BPX-70 column (Carrier gas: helium)	Gardner et al. (2017) [31]
1. Fatty acids ranging from 8:0 to 22:6 2. 2 × Ceramides; 7 × GlucosylCeramide; 22 × Phosphatidylcholine; 25 × Phosphatidylethanolamine; 5 × Phosphatidylglycerol; 2 × Phosphatidylinositol; 2 × Phosphatidylserine; Retinol; 9 × Diglycerides; 49×Triglycerideas; 11 × Sphingomyeline; 10 × Eicosanoids; 2×Cardiolipines; 10 × LysoPhosphatidylcholine/Phosphatidylethanolamine	118 participants gave samples over 24 h (each participant pooled).	−80˚C	- mL human milk ➔LLE chloroform:methanol (1:1) ➔Transesterification -	In: - Out: pooled QC (10 participants pooled samples)	1. GC–FID 30 m fused silica column 2. LC-ESI-HRMS in positive and negative mode 100 mm CSH C18 column (Solvents: acetonitrile, water, isopropanol)	Alexandre-Gouabau et al. (2018) [10]

Abbreviations: LLE liquid-liquid extraction, GC gas chromatography, FID flame ionization detector, TLC thin-layer chromatography, BHT butyrated hydroxytoluene, LC liquid chromatography, LSD light scattering detector, MS mass spectrometry, EI electron ionization, FAME fatty acid methyl ester, SPE solid phase extraction, DHA docosahexaenoic acid, AA arachidonic acid, TAG triacylglyceride, NP normal phase, HPLC high pressure liquid chromatography, ELSD evaporative light scattering detector, NMR nuclear magnetic resonance spectroscopy, MTBE methyl-tert-butyl ether, Q quadrupole, ESI electrospray ionization, APCI atmospheric-pressure chemical ionization, TOF time of flight, LTQ linear trap quadrupole, ORBI orbitrap, SPME solid-phase microextraction, SFC supercritical fluid chromatography, HRMS high resolution mass spectrometry

**Table 2:** Summary of study sampling methods and corresponding total fat content in lactating women. All studies collected pre- and post-feed samples during a 24-h period. Studies that drained entire breast for samples were excluded. Total fat reported as a range, Mean, (SD or SE) where provided (- indicates not reported or taken into account).

	Sampling		During Feed	Time of Day				Lactation Stage							
Study	i) Participant n ii) Sample n	Pre-Feed (g/L)	Post-Feed (g/L)	Morning (g/L)	Noon (g/L)	Afternoon (g/L)	Evening (g/L)	1 (g/L)	2 (g/L)	3 (g/L)	4 (g/L)	5 (g/L)	6 (g/L)	9 (g/L)	12 (g/L)
Mitoulas et al., 2002 [26]	i) 17 initially ii) 76	-	-	-	-	-	-	39.9 (SE 1.4)	35.2 (SE 1.4)	-	35.4 (SE 1.4)	-	37.3 (SE 1.4)	40.7 (SE 1.4)	40.9 (SE 3.3)
Saarela et al., 2005 [22]	i) 20 ii) 483	21.0 (SD 8.4)	57.1 (SD 4.5)	-	-	-	-	19.7 (SD 8.2)	23.5 (SD 8.8)	21.0 (SD 8.4)	16.2 (SD 9.4)	11.4 (SD 6.2)	18.8 (SD 4.2)	-	-
Jackson et al., 1988 [39]	i) 25 ii) -	0.35–21.85 (SD 1.92)	-	17.9–50.6 31.4 (SD 6.6)	-	-	20.7–45.7 31.4 (SD 6.6)	-	-	-	-	-	-	-	-
Khan et al., 2013 [23]	i) 15 ii) -	32 (SD 12)	56 (SD 17)	18.4–69.2 29.3 (SD 10.9)	22.1–80.6 35 (SD 12.9)	21.2–72 31.6 (SD 10.4)	15.9–63.3 28.1 (SD 12.2)	-	-	-	-	-	-	-	-

**Table 3:** Advantages and disadvantages of analytical instrumentation used in human milk lipidomics.

Separation/Detection Method	Advantages	Disadvantages
Gas chromatography	1. Fatty acid methyl ester analysis is well characterized 2. Flame ionisation detector is robust and easy to maintain	1. Sample derivatisation is required 2. Destructive 3. Isomers separation requires longer column and run time 4. Flame ionisation detector lacks mass selectivity
Liquid chromatography	1. No sample derivatisation required 2. Large selection of column chemistry available	1. Solvent system must be compatible with detector type
Supercritical fluid chromatography	1. No derivatisation required 2. Compatible with almost any detector type 3. Relatively inexpensive 4. Low waste output 5. Faster separation than in GC/LC 6. Higher resolution than in GC/LC	1. Polar lipid separation requires organic modifier
Thin-layer chromatography	1. Inexpensive	1. Qualitative lipid class separation only 2. Low separating resolution compared to GC and LC.
Mass spectrometry	1. High sensitivity and specificity 2. Qualitative and quantitative (with standards)	1. Expensive 2. Destructive
NMR spectroscopy	1. Non-destructive 2. Highly reproducible	1. Expensive 2. Signal overlapping in complex samples 3. Lower sensitivity than MS 4. Requires larger samples volume
